# Surveillance of Antifungal Resistance in Candidemia Fails to Inform Antifungal Stewardship in European Countries

**DOI:** 10.3390/jof8030249

**Published:** 2022-02-28

**Authors:** Liliana Galia, Maria Diletta Pezzani, Monica Compri, Astrid Callegari, Nithya Babu Rajendran, Elena Carrara, Evelina Tacconelli, the COMBACTE MAGNET EPI-Net Network

**Affiliations:** 1 Infectious Diseases Unit, Department of Diagnostics and Public Health, University of Verona, 37134 Verona, Italy; liliana.galia@univr.it (L.G.); elena.carrara@univr.it (E.C.); evelina.tacconelli@univr.it (E.T.); 2 Institute of Microbiology, Department of Diagnostics and Public Health, Azienda Ospedaliera Universitaria Integrata, 37134 Verona, Italy; monica.compri@aovr.veneto.it; 3 Department of Infectious Diseases, ASFO Santa Maria degli Angeli Hospital of Pordenone, 33170 Pordenone, Italy; callegari.astrid@gmail.com; 4 DZIF Center, Division of Infectious Diseases, Tübingen University Hospital, 72076 Tübingen, Germany; nithya.babu-rajendran@med.uni-tuebingen.de

**Keywords:** candidemia, resistance, surveillance, stewardship

## Abstract

Background: The increasing burden of candidemia and the emergence of resistance, especially among non-*Candida albicans* strains, represent a new threat for public health. We aimed to assess the status of surveillance and to identify publicly accessible resistance data in *Candida* spp. blood isolates from surveillance systems and epidemiological studies in 28 European and 4 European Free Trade Association member states. Methods: A systematic review of national and international surveillance networks, from 2015 to 2020, and peer-reviewed epidemiological surveillance studies, from 2005 to 2020, lasting for at least 12 consecutive months and with at least two centers involved, was completed to assess reporting of resistance to amphotericin B, azoles, and echinocandins in *C. albicans*, *C. glabrata*, *C. parapsilosis*, *C. tropicalis*, *C. krusei*, and *C. auris*. Results: Only 5 (Austria, Italy, Norway, Spain, and United Kingdom) of 32 countries provided resistance data for *Candida* spp blood isolates. Among 322 surveillance studies identified, 19 were included from Belgium, Denmark, Iceland, Italy, Portugal, Spain, Sweden, Switzerland, and United Kingdom. *C. albicans* and *C. glabrata* were the most monitored species, followed by *C. parapsilosis* and *C. tropicalis*. *C. krusei* was not included in any national surveillance system; 13 studies assessed resistance. No surveillance system or study reported resistance for *C. auris*. Fluconazole, voriconazole, caspofungin, and amphotericin B resistance in *C. albicans*, *C. glabrata*, and *C. parapsilosis* were the most common drug–species combination monitored. Quality of surveillance data was poor, with only two surveillance systems reporting microbiological methods and clinical data. High heterogeneity was observed in modalities of reporting, data collection, and definitions. Conclusion: Surveillance of antifungal resistance in *Candida* spp blood-isolates is fragmented and heterogeneous, delaying the application of a translational approach to the threat of antifungal resistance and the identification of proper targets for antifungal stewardship activities. International efforts are needed to implement antifungal resistance surveillance programs in order to adequately monitor antifungal resistance.

## 1. Introduction

Antifungal resistance among *Candida* species represents a serious threat for public health. Candidemia remains one of the most common leading cause of nosocomial bloodstream infections (BSI) with an attributable mortality of 30–40% despite adequate treatment [1,2]. Between 2000 and 2019, the incidence of candidemia in Europe has shown a worrisome upward trend, increasing from 2.2 cases/100,000 inhabitants/year to 3.2 cases/100,000 inhabitants/year [2]. High consumption of first-line antifungals and their inappropriate use is among the drivers of fungal resistance [3,4]. This occurs more frequently in high-risk settings, where they are routinely used for prophylaxis and or empirical treatment [5]. Hence, rising trends in resistance against azoles and echinocandines can severely impair the management of candidemia due to limited therapeutic options. The awareness of emerging resistance and species distribution is therefore of primarily importance to tailor a judicious use of the available drugs [6,7,8,9,10].

In Europe, *Candida albicans* is the most prevalent species responsible for invasive candidiasis, accounting for 70–90% of cases (with or without an identified focus) [7]. Susceptibility reports display wide geographical variabilities. For example, fluconazole non-susceptibility ranges from no cases in Iceland [11] to a 10% incidence in Spain [12]. Echinocandin resistance seems still negligible so far, being recorded in 1.4% of strains for caspofungin, 2.9% for anidulafungin, and 1.3% for micafungin, although with higher rates for anidulafungin and micafungin [13].

Of particular concern is the increased incidence of non-*Candida albicans* (NAC) strains. *Candida parapsilosis* and *Candida glabrata* are the next most common species causing invasive candidiasis, whose distribution changes at different latitudes. In fact, *C. glabrata* accounts for 9–21.1% of cases of candidemia in Northern Europe, while *C. parapsilosis* is more frequent in the Mediterranean area [8]. Many European studies [6,10,12,14,15,16,17,18,19,20,21] have shown an increase in azole resistance among invasive NAC species, although with different percentages (e.g., percentages of *C. glabrata* fluconazole resistance range from 2.8% in Iceland [11] to 88.6% in Denmark [6]).

Recently, surveillance studies have raised attention regarding the appearance of multidrug resistant (MDR) profiles among both *C. albicans* and NAC isolates [22]. In the absence of a standard definition, the term MDR for *Candida* is used to describe strains that are non-susceptible to more than one agent in more than two antifungal classes, and extensively drug resistant (XDR) is defined as non-susceptibility to more than one agent in more than three antifungal classes [22,23]. An observational study that collected data from 293 episodes of bloodstream infections (BSI) between 2001–2010 reported an incidence of 14.1% of *C. glabrata* isolates that were resistant to fluconazole and to one echinocandin [24]. Other European studies have already described the emergence of MDR strains in *C. glabrata*, *C. krusei, C. parapsilosis*, and *C. tropicalis* [15,25,26]. The global appearance of *Candida auris* as a causative agent of nosocomial outbreaks with high morbidity and mortality adds further concern to the preservation of current antifungal agents. First reported in Japan in 2009 [27], *C. auris* is intrinsically resistant to fluconazole, almost half of the isolates are resistant to amphotericin B, and it has a propensity to rapidly develop resistance to different classes of antifungals [28]. The capacity to survive for long periods in hospital environments and to colonize patients, as well as healthcare personnel, make *C. auris* a priority species to keep under surveillance [29].

Growing initiatives aimed at the implementation of data collection have been promoted by major stakeholders. In 2019, the Centers for Disease Control and Prevention (CDC) included fungal infections in the priority list of the Antibiotic Resistance Threats Report [30,31]. Moreover, in 2020, the World Health Organization (WHO) held the first meeting to develop a priority pathogen list for fungal infections of public health importance and to define research and development (R&D) priorities to encourage innovation for new strategies, drugs, and diagnostics. The Global Antimicrobial Resistance Surveillance System (GLASS) of Fungal Antimicrobial Resistance (AMR) developed an early implementation protocol focusing on invasive fungal bloodstream infections (BSIs) caused by *Candida* spp. Antifungal susceptibility data from blood *Candida* isolates, especially from patients in high-risk hospital units (e.g., intensive care units; ICU), will be available through the GLASS report [32,33,34].

The present study reviewed publicly accessible surveillance data and studies on resistant *Candida* spp. isolates in bloodstream infections (BSIs) in 28 European and 4 European Free Trade Association (EFTA) countries (pre-Brexit) to assess the status of surveillance for antifungal resistance at the European level.

## 2. Materials and Methods

### 2.1. Search Strategy

A review of publicly accessible national and international (European Centre for Disease Prevention and Control) surveillance systems and peer-reviewed epidemiological surveillance studies was carried out to comprehensively assess resistance monitoring in *Candida* spp. blood isolates. Target fungi were: *C. albicans, C. glabrata, C. parapsilosis, C. tropicalis, C. krusei*, and *C. auris*. Resistance patterns included echinocandins, azoles, and amphotericin B. The review protocol with a detailed description of the methods is available on the COMBACTE-Magnet EPI-Net website [35].

Briefly, a double-step search was adopted to map European networks. First, screening of the available catalogue of national and international surveillance systems for AMR and healthcare-associated infections (HAIs) periodically mapped by the COMBACTE MAGNET EPI-Net project [36] was performed to identify surveillance reports that provided data on resistance in invasive *Candida* species from the years 2015 onward (last updated in December 2020). An additional gray literature search using Google was run to expand the list of surveillance systems with the following search terms in the local languages of European countries: ‘Antimicrobial resistance’, ‘Antibiotic resistance’, ‘Multidrug resistance’, ‘Antifungal resistance’, ‘Candidemia’, ‘Mycosis’, ‘Fungi’, ‘Surveillance’, ‘Monitoring’, ‘Infection’, ‘Nosocomial’, ‘Healthcare’, ‘Hospital’, ‘Healthcare associated-infection’, and ‘Hospital-associated-infection.’ Systems providing data for public consultation on a periodic basis were included, as well as their most recent surveillance reports used for data extraction, while surveillance systems without publicly available information or with information older than 10 years, single center data, and reports with the same data retrieved from national surveillance systems already included (Supplementary Material Files S1, S2) were excluded.

### 2.2. Inclusion Criteria and Data Extraction

Epidemiological surveillance studies were identified through a search on PubMed using a combination of the following key terms: ‘antifungal resistance”, ‘multifungal resistance’, ‘surveillance’, ‘epidemiology’, ‘candidemia’, and ‘fungemia’. Only articles published from 1 January 2005 to 31 December 2020, conducted at least in two centers and for a minimum of 12 consecutive months, were included.

Data pertaining to the characteristics of the surveillance program, target population, samples, setting, risk factors, outcome, microbiological methodology, and CLSI or EUCAST reference guidelines as reported in the surveillance reports and studies were extracted. Data were independently collected by two reviewers and disagreements were resolved by discussion or by requesting an evaluation from a third reviewer.

### 2.3. Ethics

No ethics approval was needed for the study since the research did not involve human or animal subjects and only included data derived from published surveillance reports and epidemiology studies.

## 3. Results

### 3.1. International Surveillance Systems

Epidemiological data on resistant *Candida* species from blood cultures were not available from publicly assessable international surveillance systems in Europe.

### 3.2. National Surveillance Systems

Twenty-four EU and four EFTA countries had national surveillance systems of antibiotic resistance. The two distinct Scottish and British regional surveillance systems were considered as a unique surveillance system (UK).

**Figure 1:**
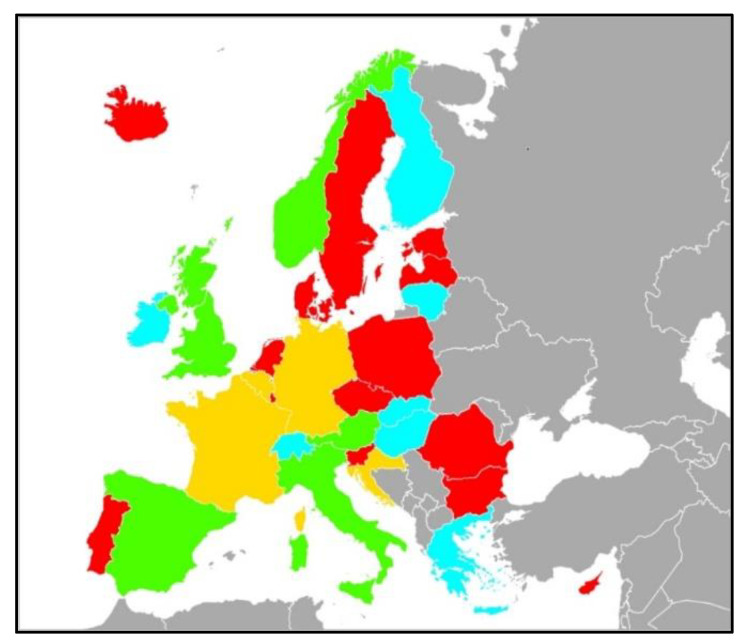
Countries with availability of surveillance data of resistance in candidemia; description of systems is provided in Supplementary Material File S3. Green: countries providing *Candida* spp. resistance data in bloodstream infections; yellow: countries providing *Candida* spp. resistance data from mixed samples (all isolates); light blue: countries provided *Candida* isolates stratified by infection type without resistance; red: countries not providing any data on resistance of *Candida* spp.

Among 52 publicly HAI and AMR national surveillance systems, Belgium, Croatia, France, and Germany provided antifungal-resistance data without stratifying by type of sample (13%). Finland, Greece, Hungary, Ireland, Lithuania, Slovakia, and Switzerland stratified *Candida* isolates by infection, but without resistance information (21%). Only five countries (16%) (Austria, Italy, Norway, Spain, and United Kingdom) reported candidemia resistance data. The remaining countries did not publish any data regarding *Candida* infections or resistance (*n* = 16, 50%) (Figure 1). Flow chart is provided in Supplementary Material File S1 and a detailed description of the surveillance systems in Supplementary Material File S3.

Regarding the species under surveillance among the five countries providing resistance data, *C. albicans* was steadily monitored; *C. parapsilosis* and *C. glabrata* were reported by four countries (Austria, Norway, Spain, and UK); *C. tropicalis* was reported by only two countries (Austria, Norway); none reported data on *C. krusei* and *C. auris*. Fluconazole, caspofungin, and amphotericin B were the antifungals most frequently assessed. Surveillance reports mainly focused on the evolution of resistance patterns to fluconazole, voriconazole, caspofungin, and amphotericin B among *C. albicans, C. glabrata*, and *C. parapsilosis* (Table 1).

All the five countries provided yearly stratified data. Three countries (Italy, Spain, and UK) measured the incidence of BSI, although with heterogeneous denominators; furthermore, only the UK distinguished candidemia incidence from the incidence of BSI caused by other pathogens. Data stratification according to clinical criteria and source of infection was rarely reported. Description of antifungal susceptibility testing (AFST), AFST interpretation (to distinguish a susceptible, intermediate, or resistant isolate), minimum inhibitory concentration (MIC) distribution (mg/L), and EUCAST or CLSI guidelines were fragmentary, as well as the number of isolates tested (in some cases less than 30) [11,14,16,17,19,37,38]. Only the Austrian and Norwegian reports provided information on MIC distribution and interpretation criteria of susceptibility results. Molecular typing of resistance, risk factors, and clinical outcomes of candidemia were not specified in any report. Antifungal consumption was rarely assessed. Table 2 summarizes published data in national reports.

### 3.3. Epidemiological Surveillance Studies

Of the 322 epidemiological surveillance studies retrieved, 133 were selected after title and abstract screening of which 19 (11 publicly funded or observational nationwide and 8 supported by pharmaceutical companies) met the inclusion criteria (flow chart in Supplementary Material File S1). European surveillance studies and description of *Candida* spp. data are listed in Supplementary Material File S4.

Regarding principal investigator driven nationwide observational studies, *C. albicans* and *C. glabrata* were the most monitored species [6,11,14,15,16,17,19,37,38,39,40], followed by *C. parapsilosis* *and C. tropicalis* [6,11,14,15,16,17,19,37,39,40]. Susceptibilities towards fluconazole, voriconazole, caspofungin, and amphotericin B were the most tracked [6,11,14,15,16,17,19,37,38,39,40]. Of note, *C. auris* was not detected in any study. Population characteristics, setting, numbers of centers and study design were reported by all studies. Results were presented as aggregate data from different years. Incidence was reported by the majority of the studies (*n* = 9, 81.8%), although using various denominators [6,11,14,15,16,17,19,39,40]. Studies adopted composite criteria to define candidemia episodes, from the first blood isolate criteria within 30 days [16,17,39] to the classification of different timeframes to determine the occurrence of a distinct candidemia episode [6,11,14,15,19,40]. Antifungal use was described only by four (36.3%) studies [6,11,14,15].

Clinical outcomes (30 day mortality) were provided only by one study from Iceland and one study from Italy [11,40]; risk factors for candidemia were described by two Italian studies and one British study [38,39,40].

All studies provided data related to species identification, laboratory methods, and reference guidelines. The number of NAC isolates diverged tested between studies, being reported in some cases fewer than 30/per year [11,14,16,17,19,37,38]. Six studies reported the MIC distribution (mg/L) and five (45.5%) provided the MIC range of fluconazole intrinsic-resistance in *C. krusei* [6,14,15,16,17,19]. Only studies from Denmark had additional information on molecular resistance typing [6,14,15]. Table 3 summarizes *Candida* species and antifungal agents targeted by epidemiological surveillance studies, compared with those supported by pharmaceutical companies. Table 4 describes the available surveillance data from epidemiological and observational nationwide studies. Combinations of monitored *Candida* spp. and antifungals are detailed in Supplementary Material File S4.

## 4. Discussion

Our study shows that, at the European level, few countries integrate antifungal resistance data for candidemia in their surveillance systems. Only Norway, Austria, and United Kingdom dedicated a section for antifungal resistance within their surveillance network for invasive infections, while Italy and Spain included this under the surveillance of HAI in ICU. From this review, the heterogeneity in species surveyed clearly emerged, as well as case definitions, antifungals monitored, and the microbiological methodology adopted for data collection and reporting.

Considering that each species has unique virulence, potential, and susceptibility profiles, precise and regular reporting of *Candida* species distribution would aid in better understanding the different epidemiological patterns between *C. albicans* and NAC specific-species. Accurate and rapid identification down to the species level is of crucial importance for the choice of appropriate treatment and ultimately for stewardship and infection prevention measures [41,42]. *C. albicans* is evenly monitored by both surveillance systems and epidemiological studies, along with *C. parapsilosis*, and *C. glabrata*. *C. krusei* mapping was covered only by epidemiological studies. Fluconazole, voriconazole, caspofungin, and amphotericin B in *C. albicans* and *C. glabrata* were the species–drug combination most frequently assessed in national surveillance reports and studies. Of note, *C. auris* was not included in any surveillance system across Europe.

We noticed a remarkable variability in case definitions among surveillance systems. Most studies considered only the first isolate of *Candida* spp. per patient, but this choice could potentially lead to underestimation of candidemia episodes and consequently the incidence of antifungal resistance. As reported by Arendrup et al. [6], the first blood isolate of *Candida* spp. is frequently obtained when exposure to antifungals is lower, particularly in case of treatment with echinocandins. From an epidemiological point of view, the first isolate is a good strategy, since the collection of multiple isolates per patient could lead to selection bias. However, this methodology might avoid the selection of strains that develop resistance under treatment. The mucocutaneous selection of antifungal-resistant *Candida* spp. has already been demonstrated in patients treated with systemic antifungals for IFI [43]. At present, the best way to select a second *Candida* spp. isolate in the same patient has not been defined, and there is still extreme variability between studies. CLSI guidelines have specified that to produce an accurate statistical estimate of cumulative bacteria susceptibility rates, the reporting of a cumulative antibiogram with ≥30 isolates tested is recommended [44]. Currently, no indications have been established for fungi susceptibility rates and the number of isolates tested is frequently <30 in both epidemiological studies and surveillance networks. The validity and representativeness of the data can be hindered by the inclusion of a low number of samples, thereby delaying the identification of relevant target for antifungal stewardship.

Differences in methodology and interpretation criteria are influenced by the constant evolution in the field of resistance identification and consequent changes in the breakpoints defined by both CLSI and EUCAST guidelines. In addition to broth microdilution methods, other standardized tests (i.e., colorimetric assays, agar-based diffusion assays and automated assays) are currently available for clinical diagnosis [34,45]. Studies in the early 2000s reported that broth microdilution was performed in accordance with EUCAST guidelines, but the interpretation was later carried out referring to CLSI criteria [46,47,48]. This choice is necessarily called into question to avoid susceptibility misclassification since it is recognized that MICs need to be interpreted by adopting the breakpoints combined with the appropriate method [34,49]. Only the Austrian and Norwegian reports clarified the microbiological test (broth microdilution and E-test, respectively) and interpretative criteria adopted in every microbiology laboratory participating in surveillance. As for bacteria, standardization of both the methodology and interpretation of in vitro data would facilitate inter-laboratory comparisons and agreement [45].

The presentation of *Candida* susceptibility patterns is thus an suitable issue that would benefit from further implementations. The GLASS protocol recommends specifying at least the AFST method used, the AFST interpretations, and MICs/zone diameters [34]. However, the reports included MIC with or without prevalence of resistance or expressed resistance as resistant/intermediate/ or susceptible strain except for the Norwegian report (S3); the Spanish, Italian studies [37,40] provided additional data such as susceptible dose-dependent (SDD)/isolates and SDD/percentage values.

This study has some limitations. We focused on European public national surveillance systems and studies, excluding outbreaks, surveillance systems, and studies that provided antifungal resistance in mixed samples and international private surveillance networks. The SENTRY and ARTEMIS-DISK, which monitors the global and European incidence of fungal infection and susceptibility, provided data from blood and pooled samples, respectively, at the continent level. International societies for human and animal mycology (ECMM, ISHAM, FUNGISCOPE) do not provide publicly available reports and consequently could not be included [50,51,52,53,54].

The consequence of the shortage of available data and heterogeneity and the difficultly in extrapolating information about MDR and XDR *Candida* spp. infections makes it problematic to promptly identify MDR strains and their distribution. It would be important to implement, within existing surveillance systems for antibiotic resistance, a module reporting figures for resistance to antifungal drugs in invasive infections. Our results also highlight the paucity of information regarding antifungal consumption, which represents another interesting cause for reflection. There is emerging literature regarding the correlation between widespread antifungal utilization, which occurs especially in vulnerable patients, and the development of resistance [3,4,55,56]. Therefore, data on consumption should be ideally tracked in parallel with antifungal resistance by active surveillance to promote better understanding of the underlying epidemiology of *Candida* spp. infections and resistance risk factors.

In 2020, the first meeting of the WHO expert Group on Identifying Priority Fungal Pathogens was held to finalize the development of a priority list of fungal pathogens of public health importance and to define related R&D priorities. The expansion of R&D coordination from bacterial infections to fungal infections underlines the dramatic increase in antifungal resistance, its associated burden, and the poor pipeline. To facilitate comparisons at a global level, standardization of surveillance system reports, including disease definition and susceptibility patterns, reporting should be prioritized. These efforts would foster the interconnection between surveillance and AFS. Improving surveillance data in the field of fungal disease is of critical importance for the appropriate definition of burden, assessment of R&D priorities, and antifungal stewardship recommendations.

## Figures and Tables

**Table 1:** Antifungals and species monitored in national surveillance systems with resistance data. For *Candida auris* and *Candida krusei* no data were available.

ANF Class	ANF Drug	*C. albicans*	*C. glabrata*	*C. parapsilosis*	*C. tropicalis*
**Azole ^1^**	**Fluconazole**	4 (AT; NO; ES; UK)	4 (AT; NO; ES *; UK)	4 (AT; NO; ES *;UK)	2 (AT; NO)
	**Itraconazole**	2 (AT; ES)	2 (AT, ES *)	2 (AT; ES *)	1 (AT)
	**Posaconazole**	1 (AT)	2 (AT; ES *)	1 (AT)	1 (AT)
	**Voriconazole**	4 (AT; NO; ES; UK)	3 (AT; ES *; UK)	4 (AT; NO; ES *;UK)	2 (AT; NO)
**Echinocandin**	**Caspofungin**	3 (AT; ES; UK)	3 (AT; ES*;UK)	3 (AT; ES*;UK)	1 (AT)
	**Anidulafungin**	2 (AT; NO)	2 (AT; NO)	2 (AT; NO)	2 (AT; NO)
	**Micafungin**	2 (AT; NO)	2 (AT; NO)	2 (AT; NO)	1 (AT)
**Polyene**	**Amphotericin B**	4 (AT; NO; ES; UK)	4 (AT; NO; ES *;UK)	4 (AT; NO; ES *;UK)	2 (AT; NO)

AT: Austria; ES: Spain; IR: intrinsic resistance; NO: Norway; UK: United Kingdom. ^1^ Italy did not specify which azoles were tested, and thus was not included in the table. * Only 2019 data are reported.

**Table 2:** National data from five European countries. Molecular typing of resistance was not reported in any of the national reports.

Characteristic Data Reporting in National Reports		Austria		Italy		Norway		Spain		UK	
		CA	NAC	CA	NAC	CA	NAC	CA	NAC	CA	NAC
Frequency of reporting (yearly)		** 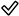 **	** 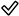 **	** 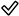 **	**--**	** 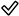 **	** 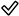 **	** 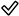 **	**--**	** 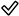 **	** 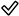 **
Data stratification (age, sex, sub-setting)		** 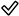 **	** 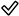 **	**--**	**--**	**--**	**--**	**--**	**--**	** 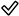 **	** 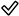 **
Source of infection		** 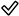 **	** 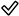 **	**--**	**--**	**--**	**--**	**--**	**--**	** 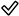 **	** 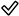 **
No. patients		** 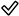 **	** 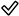 **	**--**	**--**	** 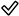 **	** 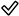 **	**--**	**--**	**--**	**--**
BSI incidence	Per 100 pt	**--**	**--**	**--**	**--**	**--**	**--**	** 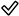 **	** 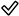 **	**--**	**--**
	Per 100 pt/7 days OR Per 100 pt/12-day catheter	**--**	**--**	** 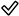 **	**--**	**--**	**--**	**--**	**--**	**--**	**--**
Candidemia incidence	Per 100,000 population	**--**	**--**	**--**	**--**	**--**	**--**	**-**	**-**	** 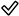 **	** 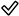 **
Total *Candida* isolates		** 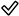 **	** 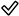 **	** 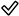 **	**-**	** 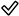 **	** 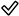 **	** 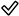 **	**-**	** 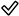 **	** 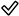 **
Specific-species identification CA /NAC		** 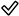 **	** 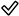 **	** 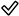 **	**-**	** 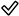 **	** 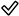 **	** 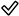 **	** 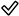 **	** 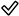 **	** 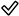 **
Antifungal consumption		**-**	**-**	**-**	**-**	**-**	**-**	** 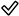 **	** 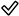 **	** 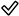 **	** 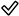 **
Laboratory method		** 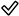 **	** 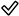 **	**NS**	**-**	** 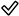 **	** 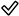 **	**NS**	**-**	**NS**	**NS**
S/SDD/R or non-susceptible		** 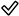 **	** 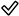 **	**NS**	**-**	** 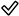 **	** 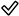 **	** 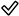 **	** 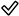 **	** 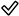 **	** 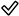 **
MIC distribution		** 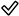 **	** 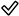 **	**NS**	**-**	** 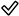 **	** 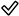 **	**NS**	**-**	**NS**	**NS**
Reference guidelines		** 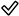 **	** 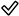 **	**NS**	**-**	** 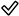 **	** 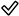 **	**NS**	**-**	** 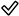 **	** 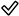 **

BSI: bloodstream infections; CA: *C. albicans*; NAC: *non-albicans Candida*; IFI: invasive fungal infections; MIC: minimum inhibitory concentration; NS: not specified; Pt: patients; R: resistant; S: susceptible; SDD: susceptible dose-dependent; dashed line: not available; 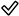: data available.

**Table 3:** Drugs and species monitored in epidemiological studies (column A) and drugs and species monitored in epidemiological studies supported by pharmaceutical companies (column B). Numbers refer to the number of countries. For *Candida auris* no data were available.

ANF Class	ANF Drug	*C. albicans*		*C. glabrata/complex*		*C. parapsilosis/complex*		*C. tropicalis*		*C. krusei*	
		A	B	A	B	A	B	A	B	A	B
**Azole**	**Fluconazole**	6 (DK;IS;IT;ES;SE; UK)	6 (BE, IT, PT, ES, CH, UK)	6 (DK;IS;IT;ES;SE; UK)	6 (BE, IT, PT, ES, CH, UK)	5 (DK;IS;IT;ES;SE;)	6 (BE, IT, PT, ES, CH, UK)	5 (DK;IS;IT;ES;SE)	5 (BE, IT, PT, ES, CH)	IR	
	**Itraconazole**	4 (DK;IS;IT;ES)	2 (ES, UK)	3 (DK;IT;ES)	2 (ES, UK)	3 (DK;IT; ES)	3 (BE, ES, UK)	3 (DK;IT;ES)	2 (BE, ES)	3 (DK; IT;ES)	2 (BE, ES)
	**Posaconazole**	2 (DK; IT)	3 (BE, PT, ES)	2 (DK; IT)	1 (ES)	2 (DK; IT)	3 (BE, PT, ES)	2 (DK; IT)	3 (BE, PT, ES)	2 (DK; IT)	1 (ES)
	**Voriconazole**	5 (DK;IT;ES;SE; UK)	5 (BE, PT, ES, CH, UK)	5 (DK;IT;ES;SE; UK)	4 (BE, ES, CH, UK)	5 (DK;IT;ES;SE; UK)	5 (BE, PT, ES, CH, UK)	4 (DK;IT;ES;SE)	4 (BE, PT, ES, CH)	4 (DK;IT;ES;SE)	3 (BE, ES, CH)
**Echinocandin**	**Caspofungin**	6 (DK;IS;IT;ES;SE; UK)	3 (IT, ES, CH)	6 (DK;IS;IT;ES;SE; UK)	3 (IT, ES, CH)	5 (DK;IS;IT;ES;SE)	3 (IT, ES, CH)	5 (DK;IS;IT;ES;SE)	3 (IT, ES, CH)	4 (DK; IT; ES; SE)	3 (IT, ES, CH)
	**Anidulafungin**	3 (DK; It; SE)	4 (BE, IT; PT, ES)	3 (DK; IT; SE)	4 (BE, IT; PT, ES)	3 (DK; IT SE)	3 (BE, PT, ES)	3 (DK; IT; SE)	4 (BE, IT; PT, ES)	3 (DK; IT; SE)	3 (IT, PT, ES)
	**Micafungin**	2 (DK; IT)	4 (BE, IT; PT, ES)	2 (DK; IT)	4 (BE, IT; PT, ES)	1 IT;	4 (BE, IT; PT, ES)	1 IT;	2(IT, ES)	1 IT;	2 (IT, ES)
**Polyene**	**Amphotericin B**	6 (DK;IS;IT; ES;SE; UK)	3 (BE, PT, ES)	6 (DK;IS;IT;ES;SE; UK)	3 (BE, PT, ES)	5 (DK;IS;IT;ES;SE)	3 (BE, PT, ES)	5 (DK;IS;IT;ES;SE)	3 (BE, PT, ES)	4 (DK;IT;ES;SE)	3 (BE, PT, ES)

BE: Belgium; CH: Switzerland; DK: Denmark; ES: Spain; IR: intrinsic resistance; IS: Iceland; IT: Italy; PT: Portugal; UK: SE: Sweden; UK: United Kingdom.

**Table 4:** Data from 11 epidemiological and observational nationwide studies. CA: *C. albicans*; NAC: *non-albicans Candida*; NS: not specified; R: resistant; SDD: susceptible dose-dependent; dash line: not available. Note: in the British study stratification by clinical criteria and incidence value were provided only for 2011 data.

Variable	Denmark (3 Studies)	Iceland (1 Study)	Italy (3 Studies)	Spain (1 Study)	Sweden (2 Studies)	UK (1 Study)
Study design	Prospective	**NS**	Prospective	Prospective	Prospective	Retrospective
	Retrospective		Retrospective		Retrospective	
Years available	2004–2009 2010–2011 2012–2015	2000–2011	2004–2005 2011–2013 2014–2015	2005–2006	2005–2006 2015–2016	2007–2011
Data presentation	Pooled	Pooled	Pooled	Pooled	Pooled	Pooled
Data stratification (age, sex, sub-setting)	** 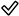 **	**-**	** 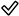 **	**-**	** 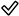 **	** 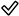 **
Incidence ^▲^	** 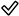 **	** 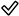 **	** 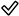 **	**-**	** 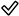 **	**-**
IFI incidence denominator	Per 100,000 inhabitants	Per 100,000 inhabitants	Per 1000 admission OR Per 100 patients	-	Per 100,000 inhabitants	-
Antifungal consumption	** 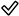 **	** 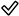 **		**-**	**-**	**-**
Episodes definition	^🞼^	^🞼^	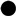	-	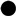	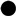
			^🞼^			
Number of patients	** 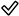 **	** 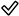 **	** 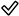 **	** 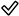 **	** 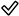 **	** 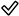 **
Total *Candida* isolates	** 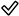 **	** 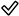 **	** 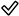 **	** 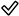 **	** 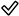 **	** 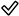 **
Species identification CA/NAC	** 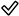 **	** 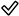 **	** 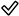 **	** 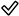 **	** 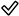 **	** 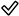 **
Molecular typing resistance	** 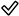 **	**-**	**-**	**-**	**-**	**-**
Laboratory method	** 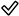 **	** 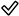 **	** 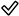 **	** 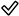 **	** 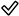 **	** 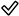 **
S/SDD/R or Non susceptible	** 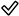 **	** 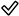 **	** 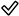 **	** 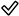 **	**NS**	** 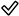 **
MIC distribution	** 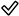 **	**NS**	** 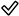 **	**NS**	** 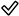 **	**NS**
Reference guidelines	** 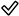 **	** 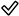 **	** 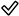 **	** 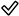 **	** 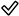 **	** 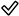 **

^▲^ Incidence of candidemia OR other fungal infections (*Cryptococcus* spp., *Fusarium* spp., *Geotrichum* spp., *Rhodotorula* spp., *Saccharomyces* spp. species). ^
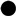
^ First blood isolate criteria within 30 days; **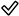:** data available. ^🞼^ Onset of new candidemia episode defined as occurring after 30 days OR isolation of a different species after 10 or 21 days.

## Data Availability

Data are available on the EPI-Net website: https://epi-net.eu/ (accessed on 30 January 2022).

## Supplementary Materials

The following are available online at https://www.mdpi.com/article/10.3390/jof8030249/s1.
