# Supplementary material for: Surveillance of Antifungal Resistance in Candidemia Fails to Inform Antifungal Stewardship in European Countries
Source: J Fungi (Basel). 2022 Feb 28;8(3):249. doi: 10.3390/jof8030249 (PMC8950249; doi:10.3390/jof8030249)
Supplement: Supplementary file 1 [file jof-08-00249-s001.zip › Supplementary material file S.3 .pdf]

S.3. Supplementary material , Epi-net links overview of European Candidemia resistance data availability (last update 31.12.2020)

Country	Acronym	Link 1	Link 2	Data available
**AMR national and international surveillance systems**				
All countries	EARS-Net	https://ecdc.europa.eu/en/surveillance-atlas-infectious-diseases	https://ecdc.europa.eu/en/about-us/partnerships-and-networks/disease-and-laboratory-networks/ears-net	Not found
All countries	FWD-Net	https://ecdc.europa.eu/en/about-us/partnerships-and-networks/disease-and-laboratory-networks/fwd-net		Not found
Austria	AURES	http://www.aures.at/		Yes
Belgium	WIV-ISP*	http://www.nsih.be/nsih/nsih_nl.asp		Candida mixed isolates and resistance (all isolates)
Croatia	ISKRA	http://www.iskra.bfm.hr/ENG/	http://www.iskra.bfm.hr/hrv/Resistance.aspx?id=66	Candida mixed isolates and resistance (all isolates)
Denmark	DANMAP	https://www.danmap.org/Downloads/Reports.aspx		Not detailed
Estonia	NAKISe	https://www.terviseamet.ee/et/nakkushaigused-menuu/tervishoiutootajale/antimikroobne-resistentsus		Not found
Finland	FIRE	https://www.thl.fi/en/web/thlfi-en/about-us/organisation/departments-and-units/infectious-diseases/bacterial-infections/fire-finnish-study-group-of-antimicrobial-resistance		Not found
France	ONERBA	http://www.onerba.org/		Not found
Germany	ARS	https://ars.rki.de/Content/Database/ResistanceDevelopment.aspx	https://ars.rki.de/Default.aspx	Not found
Germany	SARI	http://sari.eu-burden.info/		Not found

Germany	GERMAP	http://www.bvl.bund.de/DE/09_Untersuchungen/01_Aufgaben/03_Nationales%20Resistenz-Monitoring/untersuchungen_NatResistenzmonitoring_node.html		Candida mixed isolates and resistance (all isolates)
Greece	GSSAR	http://www.mednet.gr/whonet/		Candida blood isolates, No data on resistance
Hungary	NNSR*	http://www.oek.hu/oek.web		Candida isolates, No data on resistance
Iceland	Directorate of health	https://www.landlaeknir.is/smit-og-sottvarnir/syklalyfjaonaemi-syklalyfjanotkun/		Not found
Ireland	HPSC	http://www.hpsc.ie/	https://www.hpsc.ie/a-z/microbiologyantimicrobialresistance/infectioncontrolandhai/candidaauris/	Candida blood isolates, No data on resistance
Italy	AR-ISS	http://www.epicentro.iss.it/focus/resistenza_antibiotici/		Not found
Lithuania	NVSPL	http://www.hi.lt/lt/antimikrobinio-atsparumo-ataskaitos.html		Not found
Netherlands	ISIS-AR	https://www.isis-web.nl/		Not found
Netherlands	MARAN	http://www.wur.nl/nl/Expertises-Dienstverlening/Onderzoeksinstituten/Bioveterinary-Research/Publicaties/MARAN-Rapporten.htm		Not found
Netherlands	NETHMAP	http://www.swab.nl/swab/cms3.nsf/viewdoc/20BCD3983B5C390AC12575850031D33D		Not found
Norway	NORM	https://www.fhi.no/hn/helseregistre-og-registre/norm/om-norm/		Yes
Norway	NORM-VET	http://www.vetinst.no/overvaking/antibiotikaresistens-norm-vet		Yes
Portugal	ARSIP*	http://www.dgs.pt/em-destaque/programa-de-prevencao-e-controlo-de-infecao-e-resistencia-aos-antimicrobianos.aspx		Not found
Romania	CARMIN-ROM*	http://www.cnscbt.ro/index.php/		Not found
Spain	**JIACRA**	http://www.resistenciaantibioticos.es/es/publicaciones?tt=&ds=&sl=All&bd%5Bvalue%5D%5Bdate%5D=&ed%5Bvalue%5D%5Bdate%5D=		Not found

Slovenia	SKUOPZ	http://www.imi.si/strokovna-zdruzenja/skuopz/dokumenti/	http://www.imi.si/strokovna-zdruzenja/skuopz	Not found
Slovenia	NLZOH	https://www.nijz.si/sl/epidemiolosko-spremljanje-nalezljivih-bolezni-letna-in-cetrtletna-porocila		Not found
Sweden	SWEDRES	http://www.sva.se/en/antibiotics/svarm-reports		Not found
Switzerland	ANRESIS	http://anresis.ch/index.php/Interactive-database.html	http://anresis.ch/	Not found
United Kingdom (Wales)	WHAIP*	http://www.wales.nhs.uk/sites3/home.cfm?orgid=379		Not found
United Kingdom (Scotland)	HPS*	http://www.hps.scot.nhs.uk/	https://www.hps.scot.nhs.uk/a-to-z-of-topics/antimicrobial-use-and-resistance/	Yes
United Kingdom (England)	PHE*	https://www.gov.uk/government/organisations/public-health-england	https://www.gov.uk/government/publications/candidaemia-annual-data-from-voluntary-surveillance	Yes
United Kingdom (Northern Ireland)	PHA	http://www.publichealth.hscni.net/directorate-public-health/health-protection		Not found
**HAI National and international surveillance systems**				
All countries	HAI-Net	https://ecdc.europa.eu/en/about-us/networks/disease-networks-and-laboratory-networks/hai-net-about		Candida blood isolates, No data on resistance
Austria	ANISS	https://www.meduniwien.ac.at/hp/krankenhaushygiene/forschung-lehre/aniss-surveillance/		Not found
Belgium	WIV-ISP (ICU, SEP) *	http://www.nsih.be/nsih/nsih_nl.asp		Candida blood isolates, No data on resistance
Denmark	HAIBA	http://www.esundhed.dk/sundhedskvalitet/HAIBA/Sider/HAIBA_report.aspx		Not found
Finland	SIRO	https://www.thl.fi/fi/web/infektiotaudit/seuranta-ja-epidemiat/hoitoon_liittyvien_infektioiden_seuranta/siro-raportit		Candida blood isolates, No data on resistance

France	RAISIN	http://invs.santepubliquefrance.fr/Dossiers-thematiques/Maladies-infectieuses/Infections-associees-aux-soins/Surveillance-des-infections-associees-aux-soins-IAS/Surveillance-en-incidence	http://www.cpias-ile-de-france.fr/surveillance/reseau-rea.php	Candida mixed isolates and resistance (all isolates)
Germany	KISS	http://www.nrz-hygiene.de/surveillance/kiss/		Candida blood isolates, No data on resistance
Hungary	NNSR*	https://www.antsz.hu/felso_menu/temaink/jarvany/korhazifert		Candida isolates, No data on resistance
Italy	SNICh	http://assr.regione.emilia-romagna.it/it/ricerca-innovazione/prevenzione-antibioticoresistenza-infezioni/sorveglianza-controllo/sorveglianza-rischio-infettivo/infezioni-sito-chirurgico/infezioni-chirurgia-documenti		Not found
Italy	GiViTi	http://www.giviti.marionegri.it/SorveglianzaInfezioni.asp		Yes
Italy	SPIN-UTI	http://assr.regione.emilia-romagna.it/it/ricerca-innovazione/prevenzione-antibioticoresistenza-infezioni/sorveglianza-controllo/sorveglianza-rischio-infettivo/infezioni-TI		Not found
Italy	GiViTI, SPIN-UTI	http://assr.regione.emilia-romagna.it/it/ricerca-innovazione/prevenzione-antibioticoresistenza-infezioni/sorveglianza-controllo/sorveglianza-rischio-infettivo/infezioni-TI		Not found
Lithuania	HAI E.S.LI	http://www.hi.lt/lt/hospitalines.html		Candida spp isolates (SSI), No data on resistance
Netherlands	PREZIES	https://www.rivm.nl/Onderwerpen/P/PREZIES/Over_PREZIES	http://www.rivm.nl/Onderwerpen/P/PREZIES/Incidentieonderzoek_POWI	Not found
Netherlands	SNIV	http://www.rivm.nl/Onderwerpen/S/SNIV/Incidentiemeting		Not found
Norway	NOIS-POSI	http://www.fhi.no	https://www.fhi.no/nyheter/2016/infeksjoner-etter-kirurgi-nois-posi-2015/	Not found
Portugal	ARSIP*	http://www.dgs.pt/em-destaque/programa-de-prevencao-e-controlo-de-infecao-e-resistencia-aos-antimicrobianos.aspx		Not found

Romania	CARMIN-ROM*	https://www.cnscbt.ro	https://www.cnscbt.ro/index.php/analiza-date-supraveghere/infectii-nosocomiale-1	Not found
Slovakia	NNSS	http://www.epis.sk/	https://www.epis.sk/InformacnaCast/Publikacie/VyrocneSpravy.aspx	Candida blood isolates, No data on resistance
Spain	ENVIN-UCI	http://hws.vhebron.net/envin-helics/		Yes
Switzerland	SWISSNOSO	https://www.swissnoso.ch/module/ssi-surveillance/ueber-ssi-surveillance/das-modul/		Candida isolates (SSI), No data on resistance
United Kingdom	WHAIP*	http://www.wales.nhs.uk/sites3/home.cfm?orgid=379		Candida blood isolates, No data on resistance
United Kingdom	HPS*	http://www.hps.scot.nhs.uk/haiic/index.aspx		Not found
United Kingdom	PHE*	https://www.gov.uk/government/collections/surgical-site-infection-ssi-guidance-data-and-analysis		Not found

* Common national surveillance systems between healthcare-associated infections and antimicrobial resistance.
