# Supplementary material for: Surveillance of Antifungal Resistance in Candidemia Fails to Inform Antifungal Stewardship in European Countries
Source: J Fungi (Basel). 2022 Feb 28;8(3):249. doi: 10.3390/jof8030249 (PMC8950249; doi:10.3390/jof8030249)
Supplement: Supplementary file 1 [file jof-08-00249-s001.zip › Supplementary material file S.4_28022022.pdf]

	COUNTRY /REFERENCE	SETTING /YEAR	STUDY DESIGN	INFECTIONS INCIDENCE	EPISODE DEFINITION	MYCROBIOLOGICAL TEST	REFERENCE GUIDELINES	LABORATORY	SPECIES MONITORED	ANTIFUNGAL TESTED	DOI
**1**	Sweden Klingspor (2018)	Laboratory 2015- 2016	Retrospective. Nationwide study	4,7 per 100000 inhabitants	First blood culture positive	Sensititer/E-test/Vitek2	EUCAST vers. 8.0 (2015)	local lab	*C. albicans, C. glabrata, C. parapsilosis, C. tropicalis, C. krusei, C. dubliniensis, C. lusitaniae, C. kefyr, C.magnoliae, C. pelliculosa, C.sake*	AMB, AND, FLU, VOR	10.1111/myc.12816
**2**	Sweden Ericsson (2012)	Laboratory 2005-2006	Prospective. Nationwide study	4,2 per 100000 inhabitants	First blood culture positive	E-test	EUCAST vers. 1.0 (2007)/CLSI	central lab	*C.albicans, glabrata, parapsilosis, dubliniensis, tropicalis, lusitaniae, krusei,*	AMB, CAS, FLU, VOR	10.1111/1469-0691.12111
**3**	Denmark Astvad (2018)	Hospital 2012-2015	Prospective. Nationwide study	8,4 per 100000 inhabitants	First blood culture positive Second episode >21 days /different isolate	broth microdiluition EUCAST Edef 7.3/ E-test	EUCAST	central lab	*C.albicans, dubliniensis, glabrata, krusei, parapsilosis, tropicalis, spp.*	AMB, AND, MIC, FLU, VOR	10.1128/JCM.01564-17
**4**	Denmark Arendrup (2011)	Hospital 2004-2009	prospective /retrospective. Nationwide study	8,6 per 100000 inhabitants	First blood culture positive. Second episode >21days /different isolate	broth microdiluition EUCAST Edef 7.1/Etest	EUCAST/ CLSI M27-A3 (2008)	central lab	*C.albicans, dubliniensis, glabrata, krusei, parapsilosis, tropicalis, spp.*	AMB, AND, CAS, FLU, VOR, POS, ITR	10.1128/JCM.01811-10
**5**	Denmark Arendrup (2013)	Hospital 2010-2012	Prospective. Nationwide study	9,4 per 100000 inhabitants	First blood culture positive. Secondo episode >21	broth microdiluition EUCAST Edef 7.2/E-test	EUCAST/CLSI M27-A3 (2008)	central lab	*C.albicans, dubliniensis, glabrata, krusei, parapsilosis, tropicalis, spp.*	AMB, AND, CAS, FLU, VOR, POS, ITR	10.1111/1469-0691.12212

					days/ different isolate						
**6**	Iceland<br>Asmundsdottir (2013)	Hospital 2000-2011	ND	5,7 per 100000 inhabitants	First blood culture positive<br>Second episode >30days	E-test	CLSI M27-S3 (2008)	central lab	*C.albicans, glabrata, tropicalis, dubliniensis, parapsilosis*	AMB, CAS, FLU, VOR, ITR	10.1128/JCM.02566-12
**7**	Spain<br>Florez (2009)	Laboratory (2005-2006)	prospective	-	ND	SensititreYeastOne/E-test	CLSI M27-A3 e CLSI M44-S1/ECOFF	central lab	*C.albicans, parapsilosis, tropicalis, glabrata, krusei, famata, guilliermondii*	AMB, CAS, FLU, VOR, ITR	10.1016/j.eimc.2008.09.013
**8**	Italy<br>Bassetti (2007)	Hospital 2004-2005	prospective case-control	0,1 per 100 patients	First blood culture positive	broth microdiluition CLSI 2002	CLSI 2002	local lab	*C.albicans, non-albicans*	AMB, CAS, FLU, VOR, ITR	10.1016/j.diagmicrobio.2007.01.005
**9**	Italy<br>Luzzati (2016)	Hospital 2011-2013	retrospective	-	First blood culture positive.<br>second episode >30days	Sensititre Yeast One	EUCAST (2013, v 6.1)	local lab	*C.albicans, glabrata, parapsilosis, tropicalis*	AMB, CAS, FLU	10.1007/s15010-016-0924-9
**10**	Italy<br>Prigitano (2016)	Laboratory 2014-2015	ND	1,3 per 1000 admissions	First blood culture positive<br>Second episode >10days or different isolate	Sensititre Yest One/E-test	CLSI	local lab	*C.albicans, glabrata, parapsilosis, tropicalis, krusei*	AMB, AND, CAS, MIC, FLU, VOR, POS, ITR	10.1007/s15010-016-0951-6
**11**	United Kingdom<br>Spiers (2018)	Laboratory<br>2007-2011	ND	-	ND	Sensititre Yeast One/Vitek	CLSI<br>M27A - M27S4	Local lab	*C.albicans, glabrata*	FLU, VOR, AMB, CAS	10.1093/mmy/myx165

Data deriving from six countries belonging to eleven epidemiological studies/observational nationwide surveys (2 Swedish, 3 Danish, 1 Icelandic, 1 Spanish, 3 Italian, 1 British).

	COUNTRY /REFERENCE	SETTING /YEAR	STUDY DESIGN	INFECTIONS INCIDENCE	EPISODE DEFINITION	MYCROBIOLOGICAL TEST	REFERENCE GUIDELINES	LABORATORY	SPECIES MONITORED	ANTIFUNGAL TESTED	DOI
**1**	Belgium Trouvè, C., et al. (2017)	Hospital 2013-2014	Prospective	0,4 per 1000 admissions	First blood culture positive Second episode >30days	broth microdilution EUCAST Edef 7.2	EUCAST vers. 7.0	central lab	C.albicans, C.glabrata, C.parapsilosi, C.tropicalis	AMB, AND, MIC, FLU, VOR, POS	10.1007/s10096-016-2841-3
**2**	Belgium Swinne, D., et al. (2009)	Hospital 2005-2006	NS	NS	NS	broth microdilution CLSI	CLSI M44-S1 (2006)	central lab	C.glabrata, parapsilosis, tropicalis, krusei, C. incospicua, C.famata	AMB, FLU, VOR, ITR	10.1017/S0950268808001763
**3**	Switzerland Orasch, C., et al. (2013)	Hospital 2004-2009	Prospective	NS	First blood culture positive	Sensititre Yeast One	CLSI 2012	central lab	C.albicans, C.tropicalis, C.parapsilosis, C.glabrata, C.krusei	CAS, FLU, VOR	10.1111/1469-0691.12440
**4**	Portugal Faria-Ramos, I., et al. (2014)	Hospital 2011-2012 (1	Prospective	0,9 per 1000 admissions	First blood culture positive	broth microdilution EUCAST Edef 7.2	EUCAST Edef 7.2	central lab	C.albicans, C.tropicalis, C.parapsilosis, C.glabrata, C.krusei	AMB, AND, MIC, FLU, VOR, POS	10.1007/s10096-014-2194-8
**5**	UK Scotland Odds, F.C., et al. (2007)	Hospital 2005-2006	Prospective	4,8 per 100000 inhabitants /years	First blood culture positive	broth microdilution EUCAST 2002-2005	CLSI M27-A	central lab	C.albicans, C.parapsilosis, C.glabrata	FLU, VOR, ITR	10.1099/jmm.0.47239-0

**6**	Spain Peman, J., et al. (2012)	Hospital 2009-2010	Prospective	0,9 per 1000 admissions	NS	SensititreYeastOne	CLSI M27-S3	local lab	C.albicans, C.tropicalis, C.parapsilosis, C.glabrata, C.krusei, C. orthopsilosis, C. lusitaniae	AMB, AND, CAS, MIC, FLU, VOR, POS, ITR	10.1093/jac/dks019
**7**	Spain Guinea, J., et al. (2014)	Hospital 2010-2011	Prospective	NS	First blood culture positive	broth microdilution EUCAST (EDef 7.1)	EUCAST vers. 5.0 e 6.1	central lab	C.albicans, C.parapsilosis complex, C. C.glabrata complex, C.tropicalis, , C.krusei	AMB, AND, MIC, FLU, VOR, POS	10.1128/AAC.02155-13
**8**	Italy Tortorano, A.M., et al. (2013)	Laboratory 2009	Prospective	1,2 per 1000 admissions	First blood culture positive	broth microdiluition EUCAST Edef 7.1/Etest	EUCAST 2008/ CLSI	central lab	C.albicans, C.tropicalis, C.parapsilosis, C.glabrata, C. krusei	AND, MIC, CAS, FLU	10.1007/s15010-013-0455-6

Data deriving from 6 countries belonged to 8 epidemiological studies supported by pharmaceutical companies (2 Belgian, 1 italian, 1 Portuguese,2 Spanish, 1 Swiss, 1 British); NS: not specified.

**Variable in epidemiological studies supported by pharmaceutical companies**	**Belgium (2 studies)**	**Italy (1 study)**	**Portugal (1 study)**	**Spain (2 studies)**	**Switzerland (1 study)**	**UK (1 study)**
Study design	Prospective	Prospective	**NS**	Prospective	Prospective	Prospective
Years available	2005-2006<br>2013-2014	2009	2011-2012	2009-2010<br>2010-2011	2004-2009	2005-2006
Data presentation	Pooled	Single	Pooled	Pooled	Pooled	Pooled
Incidence▲	☑	☑	☑	☑	**NS**	☑
Antifungal consumption	**--**	**--**	**--**	**--**	**--**	**--**
Data stratification (age,sex,subsetting)	☑	☑	☑	☑	**--**	☑
N..patients	☑	☑	☑	☑	**--**	☑
Episodes definition	◎	●	●	●	●	●
IFI Incidence denominator	Per 1.000 admission	Per 1.000 admission	Per 1.000 admission	Per 1.000 admission	**NS**	Per 100.000 inhabitants
Total *Candida* isolates	☑	☑	☑	☑	☑	☑

Specific-species identification CA /NAC	☑	☑	☑	☑	☑	☑
Molecular typing resistance	☑	--	--	☑	--	--
Laboratory method	☑	☑	☑	☑	☑	☑
S/SDD/R or NS	☑	**NS**	☑	☑	☑	☑
MIC distribution	**NS**	☑	☑	**NS**	**NS**	**NS**
Reference guidelines	☑	☑	☑	☑	☑	☑

Data deriving from 6 countries belonged to 8 epidemiological studies supported by pharmaceutical companies. CA: *C. albicans*; NAC: *non-albicans Candida*; NS: not specified; R: resistant; SDD: susceptible dose-dependent; dashed-line:not available.

▲ Incidence of candidemia OR other fungal infections (*Cryptococcus spp., Fusarium spp., Rhodotorula spp, Saccharomyces spp, Blastoschizomyces spp.* species)

◉ First blood isolate criteria within 30 days

◎Onset of new candidemia episode defined as occurring after 30 days OR isolation of a different species after 10 or 21 days

☑ Data available
