# Supplementary material for: Surveillance of Antifungal Resistance in Candidemia Fails to Inform Antifungal Stewardship in European Countries
Source: J Fungi (Basel). 2022 Feb 28;8(3):249. doi: 10.3390/jof8030249 (PMC8950249; doi:10.3390/jof8030249)

Eligibility criteria in the national surveillance systems.	
**National surveillance systems S.1**	
**Inclusion criteria**	**Exclusion criteria**
• European surveillance systems or including European data;<br>• All ages;<br>• All languages;<br>• Blood isolates;<br>• Human data<br>• *C. albicans, C. glabrata, C. parapsilosis, C. tropicalis, C. krusei, C. auris;*<br>• Resistance to azoles, echinocandins, amphotericin-B;<br>• From the year 2015 onwards;<br>• Promoted or endorsed by national or transnational health organizations and/or scientific societies and/or clinical or research centers;<br>• Providing, or with the intention to provide, data on a periodic basis;<br>• Published scientific and 'gray' literature;	• Surveillance systems that not providing information in the last 10 years.<br>• Surveillance systems supported by private companies;<br>• Surveillance reports and systems with information not publicly available;<br>• Candida isolates data assessed in mixed samples (all isolates);<br>• Animals study;<br>• Studies reporting only on 5-fluorocytosine resistance data.

<table>
<tr><td colspan="2">Eligibility criteria in epidemiological studies.</td></tr>
<tr><td colspan="2">Surveillance studies S.2</td></tr>
<tr><td>Inclusion criteria</td><td>Exclusion criteria</td></tr>
<tr><td>
• Epidemiological surveillance studies data for at least 12 months consecutive from two or more centers (hospitals, healthcare facilities, laboratories, etc.) and with at least one data collection starting from 2005
• resistance in candidemia;
• Surveillance studies supported by pharmaceutical companies;
• All ages;
• All languages;
• Blood isolates;
• Human data;
• C. albicans, C. glabrata, C. parapsilosis, C. tropicalis, C. krusei, C. auris.
• Resistance data to azoles, echinocandins,
• amphotericin-B.
• Nationwide studies supported by pharmaceutical companies (only in case of lack of national surveillance systems).
</td><td>
• Reviews;
• Candida infections different from bloodstream ones;
• Epidemiological studies that provide data from national surveillance system already included;
• Outbreak studies;
• Animal data;
• Epidemiological surveillance studies collecting data from only one center;
• Candida isolates derived from mixed samples (all isolates);
• 5-Fluorocytosine.
</td></tr>
</table>

Figure S1. Flow chart of the selection of studies and surveillance systems.

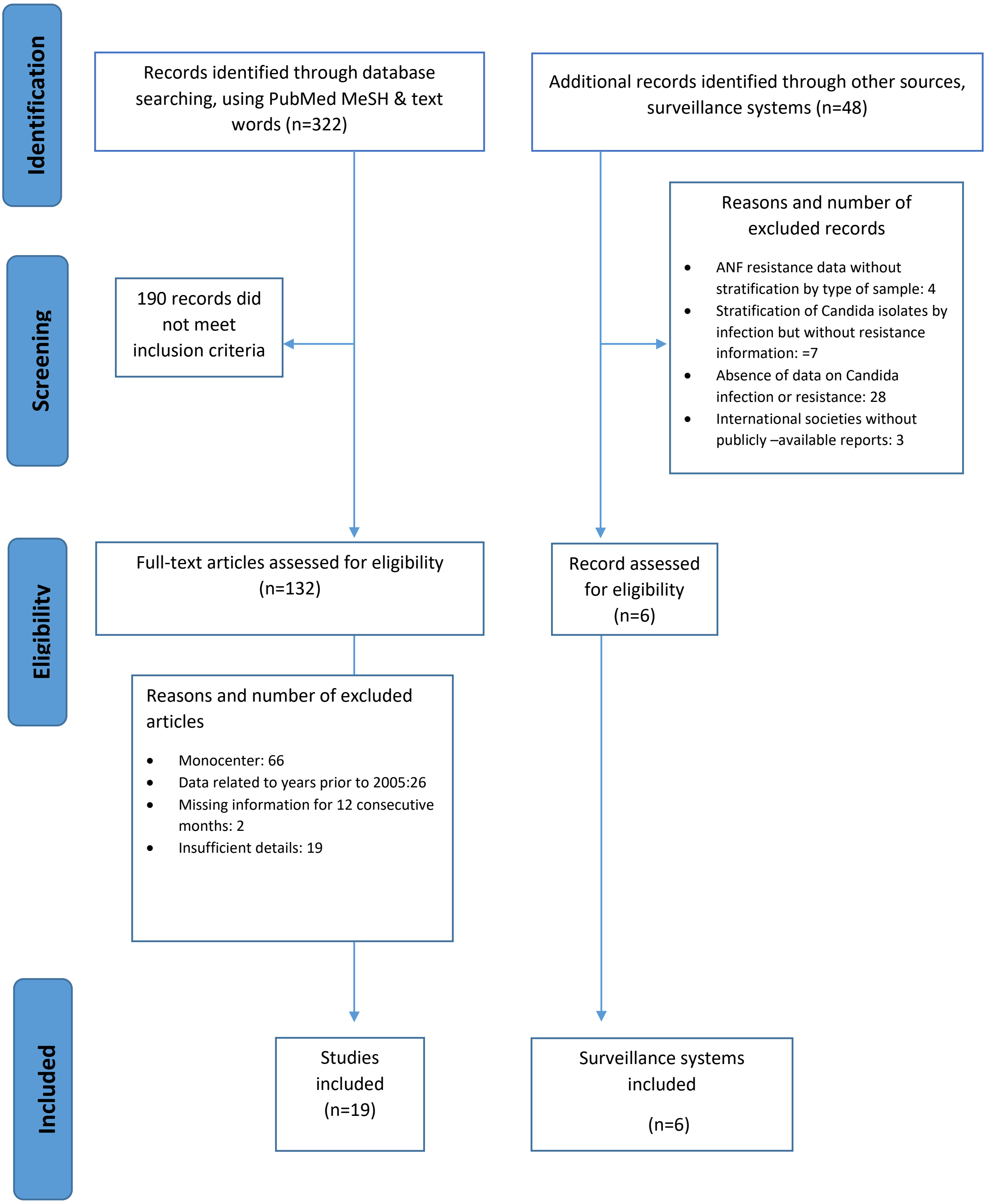

Supplement: Supplementary file 1 [file jof-08-00249-s001.zip › Supplementary material S.1 S.2_24022022_V2.pdf]
